# Continuous long-term growth of plasmacytoid dendritic cells following in vitro infection with HTLV-1

**DOI:** 10.1186/1742-4690-8-S1-A174

**Published:** 2011-06-06

**Authors:** Kathryn S Jones, Daniel C Bertolette, Xue T Bai, Cari Petrow-Sadowski, Tao Fu, Genoveffa Franchini, Christophe Nicot, Francis W Ruscetti

**Affiliations:** 1 Basic Research Program, SAIC-Frederick, Inc., National Cancer Institute-Frederick, Frederick, Maryland, 21702, USA; 2 Cancer and Inflammation Program, National Cancer Institute-Frederick, Frederick, Maryland, 21702, USA; 3 Department of Pathology and Laboratory Medicine, University of Kansas Medical Center, Kansas City, Kansas, 66160, USA; 4 Animal Models and Retroviral Vaccines Section, National Cancer Institute, Bethesda, Maryland, 20892, USA

## 

Plasmacytoid dendritic cells (pDCs) isolated from HTLV-1-infected individuals express viral proteins and can infect CD4+ T cells in vitro [1], and the proviral load in pDCs parallels the PBMC proviral load in infected individuals [2], suggesting a role for these cells in HTLV-1 transmission and persistence. We recently generated immortalized cell lines from primary pDCs infected with HTLV-1.

Peripheral blood pDCs can survive in ex vivo culture for a limited time in the presence of IL-3. Following infection with cell-free virus, pDC were cultured with activated CD4+ T cells, and separated by immunomagnetic beads. Cells capable of continuously proliferating in the absence of IL-3 were isolated and characterized. FACS analysis revealed that these cells are phenotypically similar to pDCs (CD123+, BDCA2+, CMKLR1+, CD3-, CD19-, CD14-). Preliminary studies suggest that these cells spontaneously produce cytokines and chemokines expressed by pDCs. These pDC-like cells produce high levels of HTLV-1, as determined by ELISA analyses of culture supernatants and transmission EM and, like freshly isolated pDCs, can efficiently transmit HTLV-1 to T cells. As previously shown for HTLV-1-infected T cells [3], expression of HTLV-1 in these pDC-like cells is markedly decreased following exposure to type 1 interferons. Studies to characterize the viral integration sites and to examine expression levels of proteins previously shown to be altered in HTLV-1-transformed T cells are currently in progress. The in vitro generation of immortalized HTLV-1-infected pDC-like cells capable to transmitting the virus to T cells support the notion that pDCs could be an in vivo reservoir for HTLV-1.
